# Middle School Students From China’s Rice Area Show More Adaptive Creativity but Less Innovative and Boundary-Breaking Creativity

**DOI:** 10.3389/fpsyg.2021.749229

**Published:** 2022-01-06

**Authors:** Wu-jing He, Wan-chi Wong

**Affiliations:** ^1^Department of Special Education and Counselling, The Education University of Hong Kong, Tai Po, Hong Kong SAR, China; ^2^Department of Educational Psychology, The Chinese University of Hong Kong, Shatin, Hong Kong SAR, China

**Keywords:** the rice theory, creativity, TCT–DP, China, cross-cultural study, collectivism, individualism

## Abstract

The present study aimed to conduct a cross-cultural comparison of creative thinking among Chinese middle school students from the rice- and wheat-growing areas in China through the lens of *the rice theory*, which postulates that there are major psychological differences among the individuals in these agricultural regions. Differences in cultural mindsets and creativity between the rice group (*n* = 336) and the wheat group (*n* = 347) were identified using the Chinese version of (1) the Auckland Individualism and Collectivism Scale (AICS) and (2) the Test for Creative Thinking–Drawing Production (TCT–DP), respectively. Interesting findings were obtained. The results of latent mean analyses indicate that the rice group showed significantly more collectivism and adaptive creativity than the wheat group but less individualism and innovative and boundary-breaking creativity. However, the two groups showed no significant differences in their overall creative performance, as reflected in the TCT–DP composite score. Moreover, results of hierarchical multiple regression analyses revealed that collectivism was positively related to adaptive creativity but negatively related to innovative and boundary-breaking creativity; however, a reverse pattern was found for individualism. These findings enrich the discourse regarding the rice theory and shed important light on the effect of culture on creativity.

## Introduction

Cultural differences in creativity have long been investigated (see Shao et al., 2019), and in such studies, creativity has commonly been conceptualized as a combination of originality and appropriateness (Sternberg and Lubart, 1999). Additionally, culture has often been studied according to the individualism–collectivism dimension (Tang et al., 2016). Many past studies have focused on comparisons between Western countries (representing an individualistic culture) and East Asian countries (representing a collectivistic culture) in the context of creativity (see Nouri et al., 2015). This study aimed to analyze cultural differences in creativity by examining a recently proposed perspective – the rice theory, which postulates an interesting hypothesis regarding major psychological differences among different agricultural regions in China (Talhelm et al., 2014; see also English and Geeraert, 2020).

### The Rice Theory

While culture has commonly been thought to be bounded by national borders (Hofstede, 2001), there is a debate regarding this approach of equating cultures with nations (Tung and Alain, 2010; Taras et al., 2016). Joining this discourse, the rice theory articulates that there are large cultural differences between different regions across the wide territory of mainland China. Specifically, China’s rice-growing regions show more collectivism (or interdependence culture) but less individualism (or independence culture) than its wheat-growing regions (Talhelm et al., 2014; see also Talhelm et al., 2018; Zhu et al., 2019; English and Geeraert, 2020).

Elaborating on this issue, the rice theory relates the cultural differences between the rice- and wheat-growing regions of China to two major factors of farming rice and wheat – irrigation and labor – which tend to induce different social activities related to cooperation among the individuals living in these areas (see Talhelm et al., 2014, pp. 603–604). For example, paddy rice requires a significant amount of water and massive amounts of labor (Fuller and Qin, 2009), and rice farmers need to depend on one another to (1) build and maintain effective communal irrigation systems and (2) plant and harvest rice paddy fields. As a result, cooperation is essential to growing rice; hence, people living in rice-growing areas tend to engage in intense cooperation, form tight relationships based on reciprocity, and avoid behaviors that may lead to conflict (see also English and Geeraert, 2020).

In contrast, wheat is easier to grow. First, wheat growing does not require large amounts of water (Wu et al., 2014); thus, wheat farmers can simply rely on rainfall and do not need to build irrigation networks. Second, planting and harvesting wheat requires only half the labor that rice requires (Buck, 1935), and wheat farmers can thus plant and harvest crops without relying on help from the other farmers in their regions. In this regard, intensive cooperation is much less important for growing wheat than it is for growing rice (see also English and Geeraert, 2020). Over time, communities in which individuals must cooperate intensely (e.g., rice-farming regions) tend to become more interdependent and show more characteristics of a collectivistic culture, such as valuing consensus, conformity, in-group cohesion, and harmony (Shulruf et al., 2011a; Nouri et al., 2015). In contrast, communities in which individuals do not substantially depend on one another (e.g., wheat-farming regions) tend to become more independent and show more characteristics of an individualistic culture, such as valuing exploration and openness and being autonomous and adventurous (Shulruf et al., 2011a; Nouri et al., 2015).

Importantly, the rice theory emphasizes that these principles apply to not only people who farm rice or wheat but also entire rice or wheat regions because communities that have farmed rice and wheat for years or generations may pass down the respective rice and wheat cultures; thus, the people living in these regions may inherit the corresponding culture without farming rice or wheat themselves (Talhelm et al., 2014; see also Henrich, 2014; Talhelm et al., 2018). Put differently, the theory highlights a socialization process with respect to the formation of people’s cultural mindsets in different agricultural regions, which suggests that different agricultural legacies may have influenced and will continue to influence the cultural mindsets of the people living in those areas from ancient to modern times. In this sense, rice theory can be regarded as a perspective with respect to a cultural-historical explanation of present-day psychological processes that highlights the critical roles of cultural-historical activities in the formation of the human mind, which further impacts human behaviors (see Luria, 1976; Leontiev, 1978; Vygotsky, 1997).

Lending empirical support to the rice theory, Talhelm et al. (2014) studied 1162 college students from China’s rice- and wheat-growing regions. They found that the rice group was significantly more interdependent than the wheat group. They further illustrated that China’s rice regions have several markers of a collectivistic culture, such as an interdependent self-construal, holistic thought, a high sense of loyalty to groups, low importance of the self, and a low divorce rate. Conversely, they found that the wheat regions tended to be more individualistic and exhibited more individualism, more analytic thought, stronger important of the self, more openness, and a higher divorce rate.

Consistent findings were also documented in other studies. For example, Shulruf et al. (2011a) found that while some people in China tended to exhibit more collectivistic characteristics, other people tended to display more individualistic behaviors. In a recent observational study, Talhelm et al. (2018) found that Chinese people in the wheat-growing areas exhibited more independent behaviors in cafes (e.g., sitting alone) compared to their counterparts in the rice-growing areas (Study 1). In a subsequent Study 2, Talhelm et al. (2018) conducted an interesting experiment in Starbucks around China, where they placed chairs so that they partially obstructing the aisles and observed whether people would move the chair or adjust the self to squeeze through. Again, they found empirical evidence that supported the wheat-rice difference in China with respect to people’s problem-solving styles. Specifically, people in the wheat-growing areas were more likely to solve the problem by changing the environment (i.e., moving the chairs out of the way), which is consistent with the findings that people in individualistic cultures more likely attempt to control the environment. In contrast, people in the rice-growing areas were more likely to solve the problem by changing the self (i.e., moving the self by squeezing through the chairs), which is consistent with the understanding that people in collectivist cultures tend to adjust the self to the environment (Dai and Zhao, 2021).

### Extending the Research on the Rice Theory to the Study of the Culture-Creativity Link

While Talhelm et al. (2014) proposed the rice theory and some empirical evidence has been found to support the theory (e.g., Talhelm et al., 2014, 2018), the current study aimed to extend this line of research by examining the theory with respect to the culture-creativity link. The rationale is presented below.

#### Existing Culture-Creativity Research Focused on National Comparisons and Yielded Inconsistent Findings

Creativity has been considered a cultural product and a culturally bound phenomenon (Cskiszentmihalyi, 1988; Kim, 2009). Researchers have theorized that culture may impact creativity on how much creativity can occur and the extent to which creativity can be encouraged, nurtured, and cultivated (Sternberg and Lubart, 1999). Many researchers hold the view that a collectivistic culture is less favorable toward creativity because it promotes values such as harmony, unquestioning loyalty, and conformity to group norms. These cultural values tend to inhibit the generation and expression of unique, creative ideas that constitute deviations from the norm (Ng, 2001, 2003; Nouri et al., 2015; Shao et al., 2019; Dai and Zhao, 2021). As Talhelm and English (2020) noted, collectivism emphasizes fitting in, which may be good for coping with threats but unfavorable for creativity. In contrast, an individualistic culture appears to be more favorable toward creativity because it promotes values such as exploration, openness, and being autonomous and adventurous (Nouri et al., 2015). These cultural values are thought to facilitate the expression of unique ideas and the exploration of innovative and unconventional ways to solve problems (Jackson et al., 2019; Shao et al., 2019).

However, empirical studies yielded mixed results regarding the culture-creativity link: some supported cultural differences in favor of an individualistic culture, while others showed no such differences (see Shao et al., 2019 for a review). Interestingly, a close examination of these studies suggests that most of them focused on country-level comparisons of creativity, in which creativity has primarily been compared in the context of East Asian countries (equating to a collectivistic culture) versus Western countries (equating to an individualistic culture). For example, some of these studies (Jellen and Urban, 1989; Zha et al., 2006; Yi et al., 2013) revealed that individuals from Western countries (e.g., America, England, and Germany) show more creativity than their counterparts from East Asian nations (e.g., China, India, and Indonesia). In addition, United States immigrants from individualistic countries had more patents for inventions than those from collectivistic countries (Shane, 1992). However, other studies revealed that no creativity differences could be identified in the country-level comparisons of the East and the West (e.g., Chen et al., 2002; Niu and Sternberg, 2002; Nouri et al., 2013).

#### The Rice Theory Offers an Alternative Lens to Understand the Culture-Creativity Link

Notably, in the country-level East–West comparisons regarding creativity, there appears to be a neglect of the possible influence of the potential confounding effect of many variables, including religious, ethnic, historical, political, economic, educational, and linguistic variables (Shulruf et al., 2011a; Talhelm et al., 2014; Zhu et al., 2019). Previous research has suggested that differences in these confounding variables can have a considerable influence on creativity in addition to the influence of culture with respect to the individualism–collectivism dimension (Goncalo and Staw, 2006; Kharkhurin and Motalleebi, 2008). In view of this methodological concern regarding the possible confounding effect of many variables, a more convincing test case for this issue may be a country that embraces both collectivistic and individualistic cultures while sharing a common history, government, political arena, language, and religion, as this would minimize the influence of potential confounding variables.

In this context, the rice theory offers an interesting alternative perspective that highlights that China can serve as a fitting natural test case in the study of the culture-creativity link (see also Zhu et al., 2019). While the social activities related to the different agricultural legacies of China’s rice- and wheat-growing regions may cultivate different cultural mindsets with respect to interdependence (or collectivism) and independence (or individualism), the rice- and wheat-growing regions of China are ethnically and politically unified (Talhelm et al., 2014, 2018; English and Geeraert, 2020). China is over 90% Han Chinese, and the same dynasties have ruled over both the rice and wheat regions for generations. Hanzi is the formal written language of China, and Putonghua is the official spoken language, although many dialects are spoken across different parts of China. Moreover, various belief systems, including Confucianism, Taoism, and Buddhism, coexist across China and have commonly influenced the consciousness of Chinese people for hundreds of years (Sun, 2008; Shao et al., 2019). If many of the variables that may confound the East–West comparisons are controlled, it is expected that comparing the rice- versus wheat-growing regions of China may advance the current understanding of the cultural effect on creativity.

Indeed, there is indirect empirical evidence to suggest a possible rice–wheat difference regarding Chinese people’s creativity. For example, people from rice-growing regions in China think less analytically than those from the wheat-growing regions in China (Talhelm et al., 2014; Dong et al., 2018; Zhang et al., 2021). Analytical thinking is also linked to innovation, novelty, and creativity (Witkin et al., 1977; Gorodnichenko and Roland, 2011; Henrich, 2014). Integrating these two lines of research appears to suggest a pattern that people from the rice areas would be less creative than those from the wheat areas in China. Recently, researchers have also found empirical supports to the rice theory regarding innovative thinking and innovation performance by analyzing secondary data. For example, Talhelm and English (2020) analyzed the secondary data from Chua et al. (2019) with respect to thought-style, and they found that rice-farming areas had lower innovative thinking style. Zhu et al. (2019) analyzed patent data from approximately 2000 Chinese counties and found that a legacy of wheat production was associated with more patent applications. Conversely, a legacy of rice cultivation was linked to fewer patent applications.

#### The Present Study

In this study, we aimed to extend the research of the rice theory by comparing the creative thinking of middle school students from rice-growing regions (representing a collectivistic culture) and their counterparts from wheat-growing regions (representing an individualistic culture) in China. While previous research on the rice theory predominantly focused on the adult population (e.g., Talhelm et al., 2014, 2018; Zhu et al., 2019; Talhelm and English, 2020), extending the research to an adolescent student population will help test the generalizability of the theory.

Furthermore, while there are empirical supports to the theory with respect to innovative thinking and innovation performance based on secondary data analyses (e.g., Zhu et al., 2019; Talhelm and English, 2020), we aimed to extend this line of research by using a standardized creativity test to assess the creative thinking of students. In line with the increasingly important trend in psychological studies that highlights creativity as a multi-faceted and complex construct (Dollinger et al., 2004; Wong et al., 2014), we aimed to employ a componential approach (Urban and Jellen, 2010) to conceptualize and assess creativity in this study. The componential approach emphasizes that creativity involves the contribution of the interactive synergy of multiple components, where no single component alone is sufficient to lead to a creative idea or product (Urban, 2004). Specifically, the Test of Creative Thinking–Drawing Production (TCT–DP; Urban and Jellen, 2010) was developed based on the componential model of creativity to capture various components of creativity (He and Wong, 2011). Previous research suggests that the TCT–DP consists of factorial components such as originality and adaptiveness (Lubart et al., 2010; Kâlis et al., 2014), overall meaning, boundary-breaking and unconventionality (Dollinger et al., 2004; Rudowicz, 2004). By applying the TCT–DP in the study of the rice–wheat difference in creativity, it is our intention to enhance the understanding of the culture-creativity link by analyzing the cultural effect on different aspects of creativity.

#### Hypotheses

Three hypotheses are derived according to the rice theory and relevant research:

H1: Students from China’s rice-growing areas show more collectivism but less individualism than their counterparts from China’s wheat-growing areas.

H2: Students from China’s rice-growing areas score lower on a creativity test than their counterparts from China’s wheat-growing areas.

H3: Creativity score is negatively predicted by the level of collectivism but positively predicted by the level of individualism.

## Materials and Methods

### Participants and Procedure

A total of 683 middle school students between the ages of 13 and 15 years (*M*_*age*_ = 13.9 years, SD = 0.90) were recruited from China’s rice- and wheat-growing areas. Specifically, 49.2% of the sample (*n* = 336; *M*_*age*_ = 13.8 years, SD = 0.88; 53.3% girls) was recruited from two junior secondary schools in a county within the rice areas located in Jiangxi Province, while the remaining 50.8% of the sample (*n* = 347; *M*_*age*_ = 13.9 years, SD = 0.91; 51.9% girls) was recruited from two other junior secondary schools in a county within the wheat areas located in Shaanxi Province. Jiangxi and Shaanxi Provinces were chosen as the research sites of the present study because more than 80% of the cultivated land within these provinces is devoted to rice or wheat paddies, respectively (see Figure 2 in Talhelm et al., 2014). Both counties are located in the rural area of a third-tier city of Jiangxi Province or Shaanxi Province. According to the statistics in the China Data Online, their respective GDP values (in 100 million yuan) were 135.20 and 133.39 in the year of data collection (i.e., 2018), and their respective population sizes at the year-end (in 10,000 persons) were 35.48 and 34.32.

All participants reported that they had been living in the corresponding areas since birth. Table 1 presents the demographic characteristics of the sample. The two groups showed no statistically significant differences in terms of age (*t* = −1.17, *p* = 0.279) or gender distribution (χ^2^ = 0.13, *p* = 0.713). Moreover, the two groups shared similar demographic backgrounds in terms of (1) academic performance, as indicated by their mid-term examination scores in Chinese, English, and mathematics (*t* values ≤ −1.19, *p*-values ≥ 0.269), and (2) their socioeconomic status, as indicated by the levels of education (*t* values ≤ 3.26, *p*-values ≥ 0.069) and occupations (χ^2^ values ≤ 3.07, *p*-values ≥ 0.692) of their parents. All participants and their parents provided informed consent. Participation was entirely voluntary. The creativity test and an instrument on collectivism and individualism were administered in a group setting with standard instructions; approximately 45 participants were tested at a time.

**TABLE 1:** Demographic characteristics of the two groups.

	Rice group (*n* = 336)	Wheat group (*n* = 347)		
Participant characteristics	Mean (±SD)	Mean (±SD)	*t*	*p*
Age (years)	13.8 (±0.88)	13.9 (±0.91)	−1.17	0.279
Education (years)	7.90 (±0.76)	7.93 (±0.86)	−0.38	0.538
**Academics (marks)**				
Chinese	74.6 (±9.27)	75.3 (±8.62)	−1.19	0.269
English	60.6 (±12.6)	61.6 (±12.0)	−1.12	0.288
Mathematics	83.0 (±9.79)	82.1 (±11.4)	1.02	0.305
**Parents’ education (years)**				
Father	11.9 (±1.90)	11.7 (±1.92)	3.26	0.069
Mother	9.99 (±1.91)	9.84 (±1.78)	1.26	0.258
	**Frequency (%)**	**Frequency (%)**	**χ^2^**	***p***
Gender			0.13	0.713
Male	157 (46.7)	167 (48.1)		
Female	179 (53.3)	180 (51.9)		
Father’s occupation			2.08	0.841
Agriculture	27 (8.00)	30 (8.60)		
Physical labor	122 (36.3)	134 (38.6)		
Business	98 (29.2)	95 (27.4)		
Professionals	56 (16.7)	50 (14.4)		
Civil service	23 (6.8)	30 (8.6)		
Unemployed	10 (3.0)	8 (2.3)		
Mother’s occupation			3.07	0.692
Agriculture	37 (11.0)	48 (13.8)		
Physical labor	138 (41.1)	140 (40.3)		
Business	95 (28.3)	89 (25.6)		
Professionals	43 (12.8)	39 (11.2)		
Civil service	6 (1.80)	10 (2.90)		
Unemployed	17 (5.1)	21 (6.1)		

### Instruments

#### The Auckland Individualism and Collectivism Scale

To determine whether students from the rice- and wheat-growing areas of China demonstrate different degrees of collectivism and individualism, the 26-item The Auckland Individualism and Collectivism Scale (AICS) (see Appendix 1 in Shulruf et al., 2011b, p. 65) was adapted and translated into Chinese using a back-translation procedure. The scale consisted of two dimensions: collectivism and individualism. Specifically, collectivism was measured with two subscales: (a) *harmony*, i.e., the tendency to avoid situations of conflict; and (b) *advice*, i.e., the tendency to obtain advice from others prior to making decisions. Individualism was measured with three subscales: (a) *uniqueness*, i.e., an individual’s tendency to define himself/herself as separate from others; (b) *responsibility*, i.e., an individual’s tendency to recognize his/her responsibility for his/her behavior; and (c) *compete*, i.e., an individual’s tendency to pursue his/her own goals above all.

Sample items of the two collectivism subscales include (a) “I sacrifice my self-interest for the benefit of my group” (harmony) and (b) “I consult my family before making an important decision” (advice). Sample items of the three individualism subscales include (a) “I enjoy being unique and different from others” (uniqueness), (b) “It is important for me to act as an independent person” (responsibility), and (c) “Winning is very important to me” (compete). The participants responded to each item on a 6-point Likert-type scale (1 = never, 6 = always) with respect to the frequency of such behaviors.

Support for the psychometric properties of the AICS has been well documented, and adequate goodness-of-fit statistics have been found with respect to its validity and reliability using various samples from multiple countries, including Italy, New Zealand, Portugal, the People’s Republic of China, Romania, South Africa, and Switzerland (Ciochina and Faria, 2009; Shulruf et al., 2011a,b; Györkös et al., 2012). The applicability of the scale in the context of Chinese samples has also been supported, and reliable internal consistency with Cronbach’s alphas ranging from 0.77 to 0.95 has been found (Shulruf et al., 2011a; Affum-Osei et al., 2019). Additionally, in the present study, reasonably good statistics were obtained in relation to the internal consistency of the scale (α = 0.79−0.82; see Table 5).

**TABLE 2:** Scoring criteria of the TCT–DP.

	Criterion	Descriptions	Score range
1.	Continuations (Cn)	Any use or extension of the six fragments	0–6
2.	Completions (Cm)	Any additions to the six continuations	0–6
3.	New elements (Ne)	Any new figures or symbols added to the drawing	0–6
4.	Connections that are made with a line (Cl)	Any physical linkages between the continuations or completions of the given fragments and the new elements	0–6
5.	Connections made to produce a theme (Cth)	Any elements or figures that contribute to a compositional theme	0–6
6.	Boundary breaking (Fragment-dependent) (Bfd)	Any uses of the small open square that is located outside of the large square frame	0–6
7.	Boundary breaking (Fragment-independent) (Bfi)	Any non-accidental drawing outside of the frame, excluding the use of the small open square	0–6
8.	Perspective (Pe)	Any inclusions of the three-dimensional compositional whole or elements	0–6
9.	Humor and affectivity (Hu)	Any expressions of humor or other emotions	0–6
10.	Unconventionality (Uc)	Consists of the four subcategories below: (a) Manipulations of the materials (b) Surreal or abstract drawings (c) Atypical combinations of figures and symbols (d) Non-stereotypical use of a certain element	0–3 0–3 0–3 0–3

**TABLE 3:** Rotated factor loadings, communalities, and results of EFA for the TCT–DP.

		Loadings			
Criterion		Factor 1	Factor 2	Factor 3	Communality
1.	Continuations (Cn)	**0.869**	0.104	−0.017	0.776
2.	Completions (Cm)	**0.840**	0.113	−0.081	0.658
3.	Connections that are made with a line (Cl)	**0.835**	0.221	−0.013	0.609
4.	Connections made to produce a theme (Cth)	**0.789**	−0.029	−0.109	0.612
5.	New elements (Ne)	−0.098	**0.788**	0.088	0.594
6.	Perspective (Pe)	0.149	**0.779**	0.099	0.502
7.	Boundary breaking (Fragment-independent) (Bfi)	−0.189	**0.772**	0.078	0.489
8.	Boundary breaking (Fragment-dependent) (Bfd)	−0.204	**0.693**	0.121	0.511
9.	Humor and affectivity (Hu)	−0.116	0.241	0.752	0.496
10.	Unconventionality (Uc)	−0.149	0.259	**0.733**	0.409

	Eigenvalue	2.829	2.447	1.263	
	% of total variance	28.3	24.5	12.6	
	Total variance			65.4%	

*Factor 1 was labeled Adaptive Creativity; Factor 2 was labeled Innovative and Boundary-breaking Creativity; and Factor 3 was labeled Humor and Unconventionality. Bold indicates factor loadings greater than 0.60. Total variance 65.4% can be printed in normal but not in bold.*

**TABLE 4:** Analysis of the measure invariance across the rice and the wheat culture.

Model	χ^2^	*df*	TLI	CFI	ΔCFI	RMSEA	ΔRMSEA
**AICS**							
Configural	1091.61	582	0.909	0.912	–	0.059	–
Metric	1032.49	588	0.908	0.910	0.002	0.062	0.003
Scalar	1050.11	598	0.902	0.909	0.001	0.064	0.002
**TCT–DP**							
Configural	80.18	38	0.931	0.928	–	0.058	–
Metric	99.50	50	0.921	0.922	0.006	0.062	0.004
Scalar	137.50	70	0.925	0.914	0.008	0.067	0.005

**TABLE 5:** Descriptive statistics and results of latent mean comparisons.

		Rice group (*n* = 336)		Wheat group (*n* = 347)				
Variable	α	*M*	SD	*M*	SD	Latent mean difference	*z*-score	*d*
**Collectivism**								
Harmony	0.82	4.15	0.74	3.97	0.63	0.11	10.1**	0.24
Advice	0.81	4.12	0.83	4.00	0.69	0.06	3.92*	0.11
**Individualism**								
Uniqueness	0.80	2.92	0.85	3.09	0.91	−0.12	−6.37**	−0.16
Responsibility	0.80	3.45	0.70	3.60	0.60	−0.09	−8.44**	−0.21
Compete	0.79	3.38	0.75	3.49	0.71	−0.06	−4.00*	−0.12
**Creativity**								
TCT–DP composite score	0.83	18.4	5.49	18.5	7.17	−0.41	−0.04	−0.01
**TCT–DP subscale scores**								
Adaptive creativity	0.84	14.0	4.50	12.7	4.63	5.42	11.9***	0.26
Innovative and Boundary-breaking creativity	0.81	2.31	2.96	3.82	5.11	−5.80	−20.1***	0.33
Humor and Unconventionality	0.80	2.13	2.62	1.99	2.27	0.24	0.58	0.04

*p < 0.05, **p < 0.01, ***p < 0.001.

#### The Test for Creative Thinking–Drawing Production

The TCT–DP (Form A, Urban and Jellen, 2010) was adapted and translated into Chinese *via* a back-translation procedure to measure the creative thinking of the students. In particular, it assesses creativity through a drawing task on an A4-sized testing sheet that contains six intriguing figural fragments: (a) a semicircle, (b) a point, (c) a 90° angle, (d) a curved line, (e) a broken line, and (f) a small open square. The drawing can be completed using any combination of the six figural fragments in a wide variety of ways, ranging from simple, conventional, and disjointed combinations to thematically complex, unconventional, integrated, and aesthetically interesting combinations (Dollinger et al., 2004). The test has been increasingly recognized as a promising instrument to assess creative thinking (Wong et al., 2014). Its psychometric properties have been reported in a number of studies (Dollinger et al., 2004; Karwowski et al., 2016), and the applicability of the instrument in the context of Chinese samples has also been supported in various studies (e.g., Rudowicz, 2004; He et al., 2013; He, 2018).

Based on the TCT–DP test manual, creative thinking was scored according to 10 criteria (see Table 2 for a summary of the scoring criteria). In the current study, we did not apply the criterion *Speed* because all the participants were allowed 15 min to complete the task in a group setting. A composite score was obtained by summing the points that were scored for each of the 10 criteria with no transformation. The possible score range of the TCT–DP is 0–66 points, with a higher score indicating better performance on the test. A rigorous rater training process was carried out using example drawings from other datasets to ensure accurate and reliable scoring. Two trained raters then scored a randomly selected sample of the TCT–DP protocols (*n* = 200), and a high score of interrater reliability (i.e., *r* = 0.97) was obtained for the TCT–DP composite score. Subsequently, one trained rater, who was blind to the objective and hypothesis of the study, scored all the TCT–DP protocols of the participating students. In the present study, reasonably good Cronbach’s alphas (α = 0.80–0.84; see Table 5) were obtained for the instrument, which were comparable to those reported in relevant previous studies (see He, 2018).

## Results

### Construct Validity of the Auckland Individualism and Collectivism Scale and the Test for Creative Thinking–Drawing Production

Prior to testing the hypotheses, it was important to examine the construct validity of the AICS and the TCT–DP in the sample of Chinese middle school students used in this study.

#### Construct Validity of the Auckland Individualism and Collectivism Scale

**FIGURE 1:**
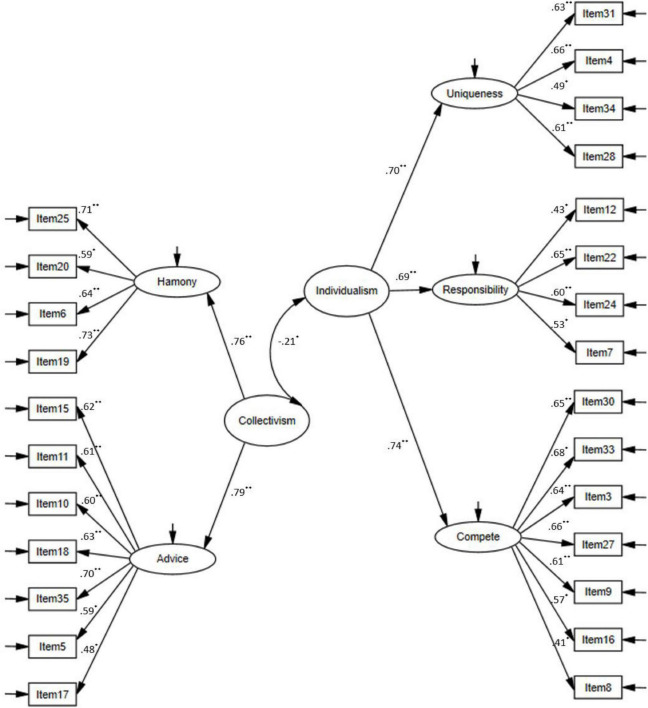
The factor structure, standardized regression weights, and correlation coefficients of the AICS; *p < 0.05; **p < 0.01; item details are available in Shulruf et al. (2011b, p. 65).

The construct validity of the AICS was analyzed using a confirmatory factor analysis (CFA) with five first-order factors (i.e., Harmony, Advice, Uniqueness, Responsibility, and Compete), and two higher-order factors (i.e., Collectivism and Individualism) were identified. Figure 1 presents the factor structure, standardized regression weights, and correlations between the factors in the model. The obtained fit indices of the resulting model (CFI = 0.912, TLI = 0.909, RMSEA = 0.059, SRMR = 0.066) were regarded as acceptable, although the χ^2^ value was statistically significant (χ^2^ = 1024.4, *df* = 293, *p* = 0.001). These results lent support to the construct validity of the scale used in the present study.

#### Factor Structure of the Test for Creative Thinking–Drawing Production

While the TCT–DP was developed based on a componential approach to creativity, which highlights the way that different components of creativity work together to contribute to the creative process (Urban and Jellen, 2010), previous studies have not shown consistent findings regarding the factor structure of this instrument (see Karwowski et al., 2016). Hence, a two-step approach involving both an exploratory factor analysis (EFA) and a CFA was used in the present study to examine the factor structure of the instrument. We randomly split the present sample into two groups to create an EFA and a CFA subsample for the respective analyses.

In the first step, an EFA was carried out with the first subsample (*n* = 341), in which the 10 TCT–DP variables were factor analyzed using the maximum likelihood method with varimax rotation. The results of Kaiser–Meyer–Olkin (KMO = 0.78) and Bartlett tests [χ^2^ (*df* = 45) = 1394.1, *p* = 0.001] supported the sampling adequacy and showed that the assumptions for the analysis were met. The results showed that three factors had eigenvalues greater than 1.0, which accounted for 65.4% of the variance. Table 3 summarizes the rotated factor loadings of the subscales and the item communalities. Factor 1 was labeled “Adaptive Creativity,” as the four components forming this factor (i.e., *Continuations*, *Completions*, *Connections made with lines*, and *Connections made to produce a theme*; see Criteria 1, 2, 4, 5 in Table 2) pertained to responses that made adaptive use of the existing elements to create an integrated or meaningful whole. Factor 2, which was called “Innovative and Boundary-breaking Creativity,” embodied four components that involved either adding *New Elements* or *Perspectives* to the provided elements or going beyond the provided box or boundary [i.e., *Boundary-breaking* (*Fragment-dependent*) and *Boundary-breaking* (*Fragment-independent*); see Criteria 3, 6, 7, 8 in Table 2]. Factor 3, which was labeled “Humor and Unconventionality,” embodied two constituents that involved (1) the expression of humor or emotions and (2) the unconventional or atypical use of existing or newly added elements (see Criteria 9 and 10 in Table 2).

**FIGURE 2:**
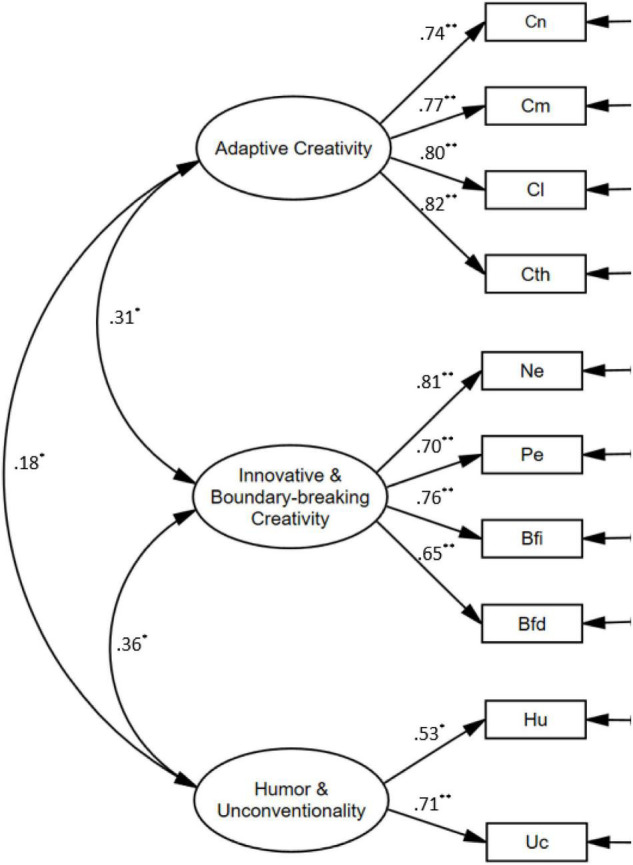
The factor structure, standardized regression weights, and correlation coefficients of the TCT–DP; *p < 0.05; **p < 0.01.

In a second step, a CFA was performed with the second subsample (*n* = 342) with the aim of replicating the three-factor model solution identified through the EFA. Figure 2 presents the factor structure of the TCT–DP, the standardized regression weights and the correlations between the factors. The results of the fit indices (CFI = 0.928, TLI = 0.931, RMSEA = 0.058, SRMR = 0.056) tended to support the construct validity of the three-factor model, although the χ^2^ value was statistically significant (χ^2^ = 76.5, *df* = 32, *p* = 0.009).

### Measurement Invariance of the Auckland Individualism and Collectivism Scale and the Test for Creative Thinking–Drawing Production

To examine whether the construct validity of the two measures was equally supported in both rice and wheat groups, measurement invariance tests were performed following progressively restrictive steps (Vandenberg and Lance, 2000). Measurement invariance was supported if the decrease in CFI and RMSEA was no greater than 0.01 and 0.015, respectively, while other fit indices such as RMSEA (<0.08) and CFI (>0.90) were acceptable (Cheung and Rensvold, 2002; Chen, 2007). As shown in Table 4, the configural invariance was supported for both AICS and TCT–DP, with all CFI values and TLI values greater than 0.90 and RMSEA values smaller than 0.60. The metric invariance for both measures was also supported after constraining the factor loadings to be equal across the two groups, where the changes in CFI and RMSEA between the configural and metric models for both AICS (ΔCFI = 0.003) and TCT–DP (ΔCFI = 0.004) were within the thresholds of 0.01 and 0.015, respectively (Cheung and Rensvold, 2002; Chen, 2007). Finally, the test results for scalar invariance revealed that the intercepts of the indicators were also invariant across the two cultural groups for both AICS (ΔCFI = 0.002) and TCT–DP (ΔCFI = 0.005), which were smaller than the thresholds of 0.01 and 0.015, respectively.

### Latent Mean Comparisons

Because measurement invariance was supported for both measures, latent mean comparisons were applied to test the hypotheses regarding cultural differences in the AICS and the TCT–DP scores. The latent mean analyses were performed by setting the wheat group as a reference group, where its latent mean was fixed to zero, and the latent mean for the rice group was freely estimated (see Steinmetz, 2018). While the latent mean of the wheat group was fixed to zero, the estimated latent mean of the rice group represents the mean difference between the two groups. Following the guidelines of Hong et al. (2003), the effect size of group differences was also computed by dividing the latent mean difference by the pooled standard deviation across the two groups. See Table 5 for the descriptive statistics and summary statistics of group comparisons.

#### Latent Mean Comparisons of the Auckland Individualism and Collectivism Scale Scores

With respect to the first Hypothesis (H1), which states that students from the rice-growing areas of China show more collectivism but less individualism than their counterparts from wheat-growing areas of China, the results reveal that the rice group scored significantly higher than the wheat group in relation to the two collectivism subscales: Harmony (latent mean difference = 0.11, *z* = 10.1, *p* = 0.003, *d* = 0.24) and Advice (latent mean difference = 0.06, *z* = 3.92, *p* = 0.04, *d* = 0.11). With reference to individualism, a reverse pattern of group differences was found, where the rice group scored significantly lower in terms of Uniqueness (latent mean difference = −0.12, *z* = −6.37, *p* = 0.008, *d* = −0.16), Responsibility (latent mean difference = −0.09, *z* = −8.44, *p* = 0.006, *d* = −0.21), and Compete (latent mean difference = −0.06, *z* = −4.00, *p* = 0.02, *d* = −0.12). Although the effect sizes of the group differences (*d* = 0.11–0.24) were small (Cohen, 1988), the results tended to support H1 with respect to the cultural differences between rice- and wheat-growing regions in China.

#### Latent Mean Comparisons of the Test for Creative Thinking–Drawing Production Scores

To test the second Hypothesis (H2), which postulates that students from China’s rice-growing areas score lower on a creativity test than their counterparts from China’s wheat-growing areas, we performed the latent mean analysis on the TCT–DP composite and factor scores to determine whether the two cultural groups would show significant differences in overall performance and different components of creative thinking. The results revealed that the rice group scored significantly lower than the wheat group in relation to only one factor score: Innovative and Boundary-breaking Creativity (latent mean difference = −5.80, *z* = −20.1, *p* = 0.001, *d* = −0.33). However, the two groups did not show a statistically significant difference in relation to the factor score Humor and Conventionality (latent mean difference = 0.24, *z* = 0.58, *p* = 0.431, *d* = 0.04). More interestingly, the results revealed a significant group difference favoring the rice group in relation to the factor score Adaptive Creativity (latent mean difference = 5.42, *z* = 11.9, *p* = 0.001, *d* = 0.26). Overall, no significant group difference was found between the students from the rice- and wheat-growing areas in terms of the TCT–DP composite score (latent mean difference = −0.41, *z* = −0.04, *p* = 0.405, *d* = −0.01). These results suggest that H2 was supported by only one TCT–DP factor (i.e., Innovative and Boundary-breaking Creativity). However, it was not supported by the other two factors (Adaptive Creativity; Humor, and Conventionality). Additionally, it was not supported by the overall creative performance of the students, as indicated by the TCT–DP composite score.

### Predicting Creativity Scores by Collectivism and Individualism

To test Hypothesis 3 (H3), which postulates that creativity score is negatively predicted by the level of collectivism but positively predicted by the level of individualism, hierarchical multiple regression analyses were conducted after the bivariate correlation analyses. The results are summarized in Tables 6, 7.

**TABLE 6:** Bivariate correlation between variables.

	1	2	3	4	5	6	7	8	9
Group (0 = wheat, 1 = rice)	1.000								
**Collectivism**									
Harmony	0.328*	1.000							
Advice	0.275*	0.517**	1.000						
**Individualism**									
Uniqueness	−0.198*	−0.067	−0.158*	1.000					
Responsibility	−0.313*	−0.064	−0.092	0.373**	1.000				
Compete	−0.218*	−0.149*	−0.131*	0.396**	0.590**	1.000			
**Creativity**									
Adaptive creativity	0.237*	0.237*	0.184*	−0.119*	−0.109	−0.092	1.000		
Innovative and Boundary-breaking creativity	−0.278*	−0.251*	−0.256*	0.373**	0.179*	0.192*	0.310*	1.000	
Humor and Unconventionality	0.030	−0.062	−0.077	0.085	0.021	0.041	0.180*	0.360*	1.000

*p < 0.05, **p < 0.01.

**TABLE 7:** Results of regression analyses.

	Adaptive creativity			Innovative and Boundary-breaking creativity			Humor and Unconventionality		
	*B*	SE	*p*	*B*	SE	*p*	*B*	SE	*p*
**Step 1**									
Gender (0 = boy, 1 = girl)	**0.168**	**0.035**	**0.032**	**−0.149**	**0.031**	**0.040**	−0.087	0.041	0.071
Age	0.042	0.036	0.363	−0.038	0.030	0.358	−0.026	0.039	0.387
Education	0.032	0.040	0.381	−0.024	0.037	0.363	−0.031	0.043	0.390
Academics									
Chinese	0.008	0.036	0.423	0.007	0.033	0.358	0.006	0.039	0.587
English	0.005	0.042	0.447	0.009	0.038	0.397	0.010	0.031	0.366
Mathematics	0.002	0.039	0.496	0.010	0.037	0.284	0.004	0.032	0.618
Parents’ education	0.093	0.031	0.138	0.112	0.034	0.052	0.098	0.035	0.058
**Step 2**									
Group (0 = wheat, 1 = rice)	**0.187**	**0.030**	**0.028**	**−0.163**	**0.037**	**0.034**	0.011	0.045	0.263
**Step 3**									
Harmony	**0.118**	**0.036**	**0.049**	**−0.123**	**0.037**	**0.047**	0.008	0.035	0.420
Advice	**0.133**	**0.031**	**0.043**	**−0.126**	**0.030**	**0.046**	0.005	0.041	0.439
Uniqueness	**−0.125**	**0.037**	**0.046**	**0.127**	**0.038**	**0.045**	0.009	0.036	0.419
Responsibility	**−0.116**	**0.037**	**0.050**	**0.117**	**0.034**	**0.050**	0.071	0.045	0.421
Compete	**−0.120**	**0.037**	**0.048**	**0.121**	**0.033**	**0.048**	0.071	0.045	0.163
**Step 4**									
Harmony × group	0.020	0.035	0.393	0.017	0.028	0.415	0.009	0.038	0.419
Advice × group	0.013	0.031	0.455	0.019	0.033	0.427	0.010	0.032	0.417
Uniqueness × group	0.017	0.029	0.423	0.012	0.030	0.460	0.008	0.030	0.400
Responsibility × group	0.021	0.033	0.390	0.015	0.034	0.449	0.011	0.039	0.460
Compete × group	0.014	0.032	0.452	0.011	0.031	0.458	0.007	0.029	0.421

*Bold indicates significant results.*

In the hierarchical multiple regression analyses, demographic variables were entered in Step 1 to control for their possible covariate effects on the creativity scores. Then, predicting variables such as the agricultural region (i.e., group), individual-level collectivism and individualism were entered in Steps 2 and 3 to understand their predictive power on creativity scores. Finally, in Step 4, the interactive terms of group and individual-level collectivism and individualism were entered to estimate if individual-level collectivism and individualism showed similar effect on the creative scores across the rice and wheat groups. The results showed that after controlling for the demographic variables, the agricultural region significantly predicted the Adaptively Creativity [*R*^2^ = 0.089, Δ*R*^2^ = 0.061, Δ*F*(2,673) = 14.7, *p* = 0.001] and Innovative and Boundary-breaking Creativity [*R*^2^ = 0.096, Δ*R*^2^ = 0.064, Δ*F*(2,73) = 15.8, *p* = 0.001]. Precisely, the rice group was positively related to Adaptive Creativity (*B* = 1.87, *p* = 0.028) but negatively related to Innovative and Boundary-breaking Creativity (*B* = −0.163, *p* = 0.034). Interestingly, the agricultural region showed no significant relationship with Humor and Unconventionality [*R*^2^ = 0.011, Δ*R*^2^ = 0.001, Δ*F*(2,673) = 1.49, *p* = 0.548].

Beyond the effect of agricultural groups, individual-level collectivism and individualism could also significantly explain additional variances of Adaptively Creativity [*R*^2^ = 0.149, Δ*R*^2^ = 0.060, Δ*F*(5,668) = 14.7, *p* = 0.003] and Innovative and Boundary-breaking Creativity [*R*^2^ = 0.167, Δ*R*^2^ = 0.071, Δ*F*(5,668) = 16.2, *p* = 0.002]. Specifically, collectivism scores were positively related to Adaptive Creativity (Harmony: *B* = 0.118, *p* = 0.049; Advice: *B* = 0.118, *p* = 0.049) but negatively related to Innovative and Boundary-breaking Creativity (Harmony: *B* = −0.123, *p* = 0.047; Advice: *B* = −0.126, *p* = 0.046). Conversely, individualism scores were negatively related to Adaptive Creativity (Uniqueness: *B* = −0.125, *p* = 0.046; Responsibility: *B* = −0.116, *p* = 0.050; Compete: *B* = −0.120, *p* = 0.048) but positively related to Innovative and Boundary-breaking Creativity (Uniqueness: *B* = 0.127, *p* = 0.038; Responsibility: *B* = 0.117, *p* = 0.034; Compete: *B* = 0.121, *p* = 0.048). Again, neither scores of collectivism nor scores of individualism showed a significant relationship with Humor and Unconventionality [*R*^2^ = 0.018, Δ*R*^2^ = 0.007, Δ*F*(5,668) = 1.77, *p* = 0.489]. Interestingly, no significant interaction effect was found for any collectivism or individualism variable on Adaptively Creativity [*R*^2^ = 0.150, Δ*R*^2^ = 0.001, Δ*F*(5,668) = 0.702, *p* = 0.622], Innovative and Boundary-breaking Creativity [*R*^2^ = 0.169, Δ*R*^2^ = 0.002, Δ*F*(5,668) = 0.811, *p* = 0.598], and Humor and Unconventionality [*R*^2^ = 0.019, Δ*R*^2^ = 0.001, Δ*F*(5,668) = 0.669, *p* = 0.713]. Altogether, these results suggest that H3 was partially supported for the creativity components such as Adaptive Creativity and Innovative and Boundary-breaking Creativity. However, it was not supported for the component Humor and Unconventionality. This pattern was similar across the rice and wheat groups.

## Discussion

The present study examined a recently proposed perspective (i.e., the rice theory; Talhelm et al., 2014) on cultural differences in creativity. Specifically, we compared the creative thinking of Chinese middle school students from China’s rice- and wheat-growing areas. Interesting findings were obtained. Although the results of the group comparisons of this study lent empirical support to the rice theory by showing that students from China’s rice-growing areas exhibit more collectivism and less individualism than those from the country’s wheat-growing areas, inconsistent findings were obtained with respect to the corresponding culture-creativity patterns, which were only partially in line with the predictions of the rice theory.

Specifically, the results of analyses of the examined students’ TCT–DP subscale scores revealed that these students from China’s rice- and wheat-growing regions had distinct creative thinking profiles. For example, the students from the rice-growing region showed superiority in adaptive creativity, which was indicated by their performance in relation to four TCT–DP scoring criteria [i.e., Continuations (Cn), Completions (Cm), Connections made with lines (Cl), and Connections made to produce a theme Completion (Cth)]. These criteria assess individuals’ abilities related to producing a complete compositional creative thought/product by making adaptive use of existing elements and components (Urban and Jellen, 2010). They involve seeing the meaningful connections and relations among a set of available elements and combining these elements in adaptive and meaningful ways. In this context, such abilities may reflect a holistic and interdependent thinking style. According to the rice theory, holistic and interdependent thinking are associated with rice culture (Talhelm et al., 2014).

In contrast, the students from the wheat-growing region performed better in innovative and boundary-breaking creativity, which was indicated by their performance in relation to the other four TCT–DP scoring criteria [i.e., New elements (Ne), Perspective (Pe), Boundary-breaking (Fragment-independent; Bfi), and Boundary-breaking (Fragment-dependent; Bfd)]. These criteria correspond to abilities related to exploration, adventures, and “thinking outside the box” (Urban and Jellen, 2010). Such abilities may be deemed to reveal an independent and individualistic thinking style. According to the rice theory, independent and individualistic thinking are characteristics of wheat culture (Talhelm et al., 2014). Overall, these findings with respect to group differences in adaptive creativity (favoring the rice group) and innovative and boundary-breaking creativity (favoring the wheat group) are in accordance with the rice theory, which posits that there are major psychological differences between the rice- and wheat-growing cultures of China. While previous research has shown that people from the rice and wheat cultures in China display substantial differences in terms of cultural thought (Talhelm et al., 2014), problem-solving style (Talhelm et al., 2018), analytical thinking (Dong et al., 2018), innovative thinking (Talhelm and English, 2020), and innovation performance (Zhu et al., 2019), we extended this line of research by showing that middle school students from the rice- and wheat-growing areas in China also exhibited different creative thinking patterns.

Notably, there were no differences between the two groups in relation to the creativity component of the TCT–DP that assesses humor and unconventionality. Moreover, the results of an analysis of the TCT–DP composite score did not indicate a statistically significant difference between the two groups, which suggests that although the two groups showed some differences in relation to creative thinking patterns, they did not show differences in relation to all the examined creativity components or in relation to their overall creative thinking performance. This finding seems to be inconsistent with those of earlier cross-cultural studies on creativity, which suggested a difference favoring individualistic cultures (e.g., Jellen and Urban, 1989; Zha et al., 2006; Yi et al., 2013). These studies found that the rice and wheat regions of mainland China resemble collectivistic and individualistic cultures, respectively (Talhelm et al., 2014), and some empirical evidence has suggested that collectivism tends to impede creativity while individualism is more likely to foster creativity (Goncalo and Staw, 2006; Nouri et al., 2015). Therefore, it is notable that the present study showed that individuals from the wheat-growing areas of China (which are thought to show more characteristics of an individualistic culture) did not demonstrate an overall creative thinking performance that was superior to that of those from the rice-growing areas (which are thought to show more characteristics of a collectivistic culture).

One possibility is that both individualism and holistic thinking are important in the context of creativity and contribute differently to the overall creative thinking performance of individuals. Indeed, our hierarchical multiple regression analysis results reveal that collectivism scores were positively related to adaptive creativity but negatively related to innovative and boundary-breaking creativity. A reverse pattern was found for individualism, which showed a negative relationship with adaptive creativity but a positive relationship with innovative and boundary-breaking creativity. Our findings appear to be consistent with those reported by Yao et al. (2012), who showed that individualism and collectivism relate differently to different stages of the creative process. While individualism was shown to foster idea generation, collectivism was shown to promote idea implementation. These authors posited that individualists perform better in relation to idea generation because they are more open to new experiences, autonomous, self-confident, and impulsive. However, collectivists are more adaptive during the implementation stage because they tend to be interdependent, sociable, and cooperative. Hence, the empirical data derived from our study and from that of Yao et al. (2012) consistently suggest that individuals from rice/collectivistic cultures are not necessarily less creative than those from wheat/individualistic cultures. Rather, individuals from these two regions have respective strengths and weaknesses in relation to different aspects of creative thinking.

In the interpretation of the findings, it is important to highlight some limitations of this research and propose future research directions accordingly. First, the sample of this study consisted of only junior secondary students between the ages of 13 and 15 years; thus, the sample was quite homogenous in terms of age range and educational background. Future research should extend this investigation to more heterogeneous samples that consist of individuals of different age groups and educational backgrounds. Nonetheless, we must be mindful that samples of university students and adult workers might involve migrant populations. Taking into account the nature of the present study, which focused on comparing the creative thinking of Chinese students from the country’s rice- and wheat-growing regions, we considered it more appropriate to recruit participants from rural areas rather than cities, given the possibility that a city population might be include migrants from various agricultural areas. Based on the county-level sampling in rural areas, the results in the present study regarding creativity scores might not be generalizable to Chinese students living in cities. Furthermore, it should be noted that the sampling in this study involved only one province and two schools in a rice region and a wheat region, respectively. Notwithstanding this limitation, the current study can be considered an initial study that invites other studies to replicate its work in more provinces in rice- and wheat-growing regions. Moreover, because educational resources and examination practices of different schools in different provinces may be different, future research should also address the issue involving school effects.

Further limitations involve the approach to addressing the complexity of cultural issues. As with most cross-cultural studies on creativity, the current study adopted definitions of creativity that originate from Western countries. However, it has been suggested that creativity is at least partially culturally specific; thus, a culturally specific definition of creativity should be taken into consideration (Rudowicz and Hui, 1997). The development of an indigenous definition and a measurement of creativity that is specific to Chinese culture is a challenging task for future creativity studies in the Chinese context. With respect to the use of the AICS, only one aspect of cultural differences (i.e., collectivism–individualism) was addressed in this study. Future research should consider the complexity and multi-dimensionality of culture. The final remark concerns the theoretical perspective. While using the rice theory as a lens to examine creative thinking is a valuable approach, it is not the only lens that enables studying psychological differences from a cultural-historical perspective. Future studies could consider a further theoretical lens (e.g., sociocultural theories of distributed creativity; see Glãveanu, 2014) to obtain a more complete picture of the cultural influence on creativity.

Despite some limitations, this research adds nuance to previous research with respect to the culture-creativity link. In particular, two important contributions should be highlighted with reference to enriching the culture-creativity research by applying the rice theory and a creativity test using a componential approach (i.e., TCT–DP). First, through the application of the rice theory, the culture-creativity link was examined according to cross-cultural comparisons within a single country. The obtained findings added new knowledge to the existing culture-creativity literature, which has focused predominantly on cross-cultural comparisons across nations. Second, the application of the TCT–DP and the refined analysis produced detailed findings regarding cultural differences in different aspects of creative thinking. The findings suggest both strengths and weaknesses in different aspects of creative thinking associated with different cultures. Overall, the nuances of cultural influence on creativity thinking style were reflected in the present study through the applications of the rice theory and the TCT–DP.

## Data Availability Statement

The original contributions presented in the study are included in the article, further inquiries can be directed to the corresponding author.

## Ethics Statement

The studies involving human participants were reviewed and approved by the Human Research Ethics Committee of The Education University of Hong Kong. Written informed consent to participate in this study was provided by the participants’ legal guardian/next of kin.

## Author Contributions

W-JH contributed to the conception and design of the work as well as the acquisition, analysis, and interpretation of the data. Both authors contributed to the article and approved the submitted version.

## Conflict of Interest

The authors declare that the research was conducted in the absence of any commercial or financial relationships that could be construed as a potential conflict of interest.

## Publisher’s Note

All claims expressed in this article are solely those of the authors and do not necessarily represent those of their affiliated organizations, or those of the publisher, the editors and the reviewers. Any product that may be evaluated in this article, or claim that may be made by its manufacturer, is not guaranteed or endorsed by the publisher.
